# Impact of a sepsis bundle in wards of a tertiary hospital

**DOI:** 10.1186/s40560-017-0231-2

**Published:** 2017-07-18

**Authors:** F. Teles, W. G. Rodrigues, M. G. T. C. Alves, C. F. T. Albuquerque, S. M. O. Bastos, M. F. A. Mota, E. S. Mota, F. J. L. Silva

**Affiliations:** Santa Casa de Misericórdia de Maceió, Barão de Maceió Street, 288, Downtown, Maceió, Alagoas 57020-360 Brazil

**Keywords:** 3-h bundle, Sepsis protocol, Mortality, Wards

## Abstract

**Background:**

Sepsis is a prevalent disease worldwide and still exhibits high rates of mortality. In the last years, many interventions aiming a positive impact on sepsis evolution have been studied. One of the main is the use of managed care protocols (sepsis bundles), which consist in systematization of diagnosis and treatment, such as standardization of antibiotics, collection of specific tests (cultures, lactate), and fluid replacement. Some studies have shown a reduction in hospital costs and lower mortality with the use of these tools. In the present study, we evaluated the impact of a sepsis bundle in wards of a tertiary hospital.

**Methods:**

One hundred sixty-seven patients were retrospectively studied. The intervention was called “3-h bundle” and consisted of collecting lactate and cultures, start broad-spectrum antibiotics in the first hour of sepsis diagnosis, and volume replacement with crystalloid if hypotension or lactate ≥2 mmol/L.

**Results:**

The overall mortality was 31.1%. Individuals who received the 3-h bundle showed a 44% lower mortality in comparison with who did not (25.6 vs. 45.7%; *p* = 0.01). Furthermore, the use of the sepsis bundle was independently correlated with lower mortality (OR = 0.175; CI = 0.04–0.64; *p* = 0.009). Therefore, a lower need for ICU admission and shorter length of stay in these units were observed in patients who received the intervention.

**Conclusion:**

The use of a sepsis protocol with systematic care in wards was associated with lower mortality, less need for ICU admission and shorter stay on these units.

**Electronic supplementary material:**

The online version of this article (doi:10.1186/s40560-017-0231-2) contains supplementary material, which is available to authorized users.

## Background

Sepsis remains a major challenge for health professionals worldwide. In Brazil, it is estimated that 30% of beds in intensive care units (ICU) are filled by patients with this diagnosis, with an overall mortality of 55.7% [1]. Because of their high mortality, the development of interventions with a positive impact on the outcome of septic patients has been encouraged [2, 3]. One of the main is the use of managed care protocols (sepsis bundles), which consist in systematization of diagnosis and treatment, such as standardization of antibiotics, collection of specific tests (cultures, lactate), volume replacement, and vasopressors [3, 4]. Some studies have shown a reduction in hospital costs and a lower mortality with the use of these protocols in emergency rooms and intensive care units [5–8]. However, data about the impact of these protocols in wards is still scarce. This study aimed to assess the impact of a sepsis protocol on the outcomes of patients in wards of a tertiary hospital.

## Methods

We performed an observational retrospective study carried out in Osvaldo Brandão Vilela Unit (OBVU) at Santa Casa de Misericordia de Maceió Hospital, with data collection of medical records from January 2012 to December 2013. The study was preceded by approval of Research Ethics Committee of State University of Health Sciences of Alagoas (UNCISAL) (protocol number: 42247014.2.0000.5011). The OBVU is a sector for users of the public health system, who admitted patients for elective clinical or surgical treatment, and is a compound of 12 wards, with 78 beds (average of six beds for ward), predominantly, for general clinical diseases (90%), but also receive patients from oncology, general surgery, and orthopedics. OBVU has as support for critical patients one general ICU with 10 beds, one neurological ICU with 10 beds, and two cardiac ICUs (10 beds each). Individuals over 18 years old and diagnosed with sepsis were included. Sepsis was defined as the presence of infection, together with systemic inflammatory response. When followed by organ dysfunction or hemodynamic instability, the diagnosis of severe sepsis and septic shock were established, respectively [9]. Patients who had no sufficient clinical or laboratory data for analysis were excluded, as well as patients who received an incomplete 3-h bundle. The following variables were assessed: age, sex, length of stay, Charlson comorbidity index, severity score Acute Physiology and Chronic Health Evaluation (APACHE) II, medical specialty responsible for hospitalization, need for ICU, length of stay in ICU, results of cultures, death, and the possible source of infection. Abdominal sepsis was based on clinical history and examination by ultrasound, CT, or analysis of peritoneal fluid (cell count and culture). Pulmonary sepsis was based on a history of productive cough or change in sputum characteristics in patients with chronic lung disease and x-ray or chest CT scan. Comorbidities that are part of the APACHE II were defined as follows: cirrhosis by the presence of signs of chronic liver (ascites, spiders veins, gynecomastia, palmar erythema) and compatible laboratory findings (bilirubin, prothrombin time, albumin); heart failure based on clinical parameters such as jugular swelling, palpable liver, pulmonary edema, or echocardiogram findings; chronic obstructive pulmonary disease (Tiffeneau index <70%) and chronic kidney disease by a creatinine clearance <15 ml/min/1.73 m^2^ (CKD-EPI); or patients on chronic dialysis. Sepsis was considered community acquired when diagnosed up to 72 h of hospital admission and hospital acquired after this. The sepsis protocol was implemented in December 2011 and called “3-h bundle”. It consisted of (1) lactate and cultures collection (blood, urine, and catheter tip based on clinical suspicion), (2) early broad-spectrum antibiotics in the first hour of sepsis diagnosis, based on hospital infection commission recommendations [3], and (3) rapid volume replacement with crystalloid (saline 0.9%) in the event of hypotension (MAP <65 mmHg) or lactate ≥2 mmol/L in a fixed volume of 30 ml/kg bolus by peripheral venous access. A central catheter was indicated for norepinephrine infusion in patients who maintained hypotension despite the aforementioned volume expansion. Central venous pressure was not measured routinely, neither volume expansion based on this parameter. Patients requiring vasoactive drugs, in respiratory distress or with decreased level of consciousness, were transferred to ICU.

Numerical variables were expressed as mean ± standard deviation (SD) or median with interquartile range, after the Kolmogorov-Smirnov normality test. The associations between continuous variables were measured by the Student *t* test and by chi-square test for categorical variables. Some variables were compared according to mortality or receiving of 3-h bundle. All patients included in the 3-h bundle group received the complete intervention (lactate and cultures collection, antibiotics, and volume replacement). The variables that correlated with mortality in univariate analysis had their risk adjusted by logistic regression (see the “Methods” section). The significance level of *p* < 0.05 and 95% confidence interval were adopted. All statistical analysis was performed using the Statistical Package for the Social Sciences software (SPSS version 20).

## Results

Two hundred nine medical charts were analyzed, and 167 met the inclusion criteria. The general data of the sample are shown in Table 1. In relation to demographic data, mean age was 51.52 ± 20.29 years and 98 (58.7%) female. The average length of stay was 27.43 ± 19.36 days. The mean Charlson comorbidity index was 3.07 ± 2.47, and APACHE II was 10.94 ± 5.45. Patients were hospitalized for various specialties: internal medicine with 119 (71.2%) cases, general surgery 15 (9%), hematology 10 (6%), oncology 10 (6%), nephrology 6 (3.6%), cardiology 4 (2.4%), and orthopedics 3 (1.8%). Regarding the type of sepsis, 91 (54.5%) patients presented community acquired, while 76 (45.5%) nosocomial sepsis. Sepsis classification was as follows: 106 (63.5%) uncomplicated sepsis, 56 (33.5%) severe sepsis, and 5 (3.0%) with septic shock. The possible source of infection was 56 (33.5%) pulmonary, 36 (21.6%) abdominal, 27 (16.2%) urinary, 20 (12%) soft tissue, 5 (3%) wound, 1 (0.6%) central venous catheter, 10 (6%) more than one source, and 12 (7.2%) indeterminate. According to the sepsis bundle, 121 patients (72.5%) received 3-h package and the results of blood cultures were negative in 134 (80.2%) of the samples. The cultures were positive for gram-positive microorganisms in 13 (7.8%) cases and gram negative in 10 (5.9%). The most frequent agents identified were *Staphylococcus aureus* in 9 patients (39.1%) and *Escherichia coli* in 3 cases (13%). Other agents have been identified in only one culture (4.3%) each. The average lactate at diagnosis was 1.4 ± 0.78 mmol/L. There was admission to ICU in 32 patients (19.2%). The mean ICU stay was 6.53 ± 6.36 days. The overall mortality was 31.1% (52 patients), being higher in more severe forms of sepsis (50% in severe sepsis and 40% in septic shock versus 20.8% in uncomplicated sepsis; *p* = 0.001). Assessing the variables according to the occurrence of death (Table 2), we observed that more patients received the 3-h bundle on survivor group (78.3 versus 59.6% of non-survivors; *p* = 0.013). There was a higher frequency of severe sepsis in non-survivor group (53.8 versus 24.3% in survivors; *p* = 0.001). Table 3 shows the distribution of the variables according to the use of 3-h bundle. A lower mortality was observed in patients who undergone the sepsis protocol (25.6 versus 45.7% without sepsis protocol; *p* = 0.013) and shorter length of stay in ICU (9.0 ± 5.90 versus 4.6 ± 6.20 days; *p* < 0.0001). There was a tendency to greater frequency of ICU admissions in patients who did not receive the bundle (28.3 versus 15.8%; *p* = 0.06).

**Table 1:** General data of the sample (*n* = 167)

Variables	Results
Age (years)	51.52 ± 20.29
Female	98 (58.7%)
Charlson	3.07 ± 2.47
APACHE II	10.94 ± 5.45
Type of ward	
Medical clinic	119 (71.2%)
General surgery	15 (9%)
Hematology	10 (6%)
Oncology	10 (6%)
Nephrology	6 (3.6%)
Cardiology	4 (2.4%)
Orthopedics	3 (1.8%)
Nosocomial sepsis	76 (45.5%)
Sepsis classification	
Sepsis	106 (63.5%)
Severe sepsis	56 (33.5%)
Septic shock	5 (3.0%)
Sepsis origin	
Lung	56 (33.5%)
Abdominal	36 (21.6%)
Urinary tract	27 (16.2%)
Soft tissue	20 (12%)
Surgical wound	5 (3%)
Catheter	1 (0.6%)
Unknown	12 (7.2%)
3-h bundle	121 (72.5%)
Blood cultures	
Negative	134 (80.2%)
Gram positive	13 (7.8%)
Gram negative	10 (5.9%)
Length of stay (days)	27.43 ± 19.36
Management in ICU	32 (19.2%)
Death	52 (31.1%)

Results expressed as mean ± standard deviation or absolute number and percentage

**Table 2:** Distribution of variables according to mortality

Variables	Survivors (*n* = 115)	Non-survivors (*n* = 52)	*p*
Age (years)	49.14 ± 20.44	56.76 ± 19.11	0.02
Female	71 (61.7%)	27 (51.9%)	0.23
Nosocomial sepsis	49 (42.6%)	27 (51.9%)	0.26
Sepsis classification			0.001
Sepsis	84 (73.0%)	22 (42.3%)	
Severe sepsis	28 (24.3%)	28 (53.8%)	
Septic shock	3 (2.6%)	2 (3.8%)	
Sepsis origin			0.65
Lung	39 (33.9%)	17 (32.7%)	
Abdominal	22 (19.1%)	14 (26.9%)	
Urinary tract	20 (17.4%)	7 (13.5%)	
Soft tissue	12 (10.4%)	8 (15.4%)	
Surgical wound	3 (2.6%)	2 (3.8%)	
Catheter	1 (0.9%)	–	
Unknown	9 (7.8%)	3 (5.8%)	
3-h bundle	90 (78.3%)	31 (59.6%)	0.01
APACHE II	10.45 ± 5.16	12.37 ± 6.09	0.04
CHARLSON	2.71 ± 2.31	3.88 ± 2.64	0.004
Lactate	1.38 ± 0.81	1.59 ± 0.64	0.10
ICU management	11 (9.7%)	21 (40.4%)	0.0001

Results expressed in absolute number and percentage. *APACHE* acute physiology and chronic health evaluation

**Table 3:** Distribution of variables according to the use of 3-h bundle

Variables	Bundle (*n* = 121)	No bundle (*n* = 46)	*p*
Age (years)	53.19 ± 20.50	47.13 ± 19.25	0.08
Female	76 (62.8%)	22 (47.8%)	0.08
Stay in hospital (days)	26.41 ± 18.74	30.13 ± 20.90	0.26
Charlson	3.16 ± 2.49	2.84 ± 2.44	0.46
APACHE II	11.10 ± 5.40	10.13 ± 5.80	0.29
Stay in ICU (days)	4.64 ± 6.20	9 ± 5.90	<0.0001
Lactate	1.44 ± 0.80	1.40 ± 0.61	0.75
Nosocomial sepsis	58 (47.9%)	18 (39.1%)	0.30
Sepsis classification			0.19
Sepsis	80 (66.1%)	26 (56.5%)	
Severe sepsis	39 (32.2%)	17 (37.0%)	
Septic shock	2 (1.7%)	3 (6.5%)	
Sepsis origin			0.07
Lung	41 (33.9%)	15 (32.6%)	
Abdominal	29 (24.0%)	7 (15.2%)	
Urinary tract	21 (17.4%)	6 (13%)	
Soft parts	12 (9.9%)	8 (17.4%)	
Surgical wound	4 (3.3%)	1 (2.2%)	
Catheter	–	1 (2.2%)	
Unknown	5 (4.1%)	7 (15.2%)	
Management in ICU	19 (15.8%)	13 (28.3%)	0.06
Death	31 (25.6%)	21 (45.7%)	0.01

In the logistic regression model (Table 4), the only risk factor independently correlated with mortality was the use of 3-h package (OR = 0.175, CI = 0.047 to 0.646, *p* = 0.009). Other variables tested (age, Charlson comorbidity index, APACHE II, and severity of sepsis) had no independent correlation with death.

**Table 4:** Independent risk factors for mortality (logistic regression)

Variables	OR	CI	*p*
Age	1.016	0.980–1.053	0.39
Charlson	1.129	0.833–1.532	0.43
APACHE II	1.019	0.918–1.131	0.72
Sepsis classification	1.891	0.701–5.103	0.20
3-h bundle	0.175	0.047–0.646	0.009

*OR* odds ratio, *CI* confidence interval

## Discussion

Our study evaluated patients in wards, most with uncomplicated sepsis and with a mean APACHE II score of 10.9. However, the overall mortality (31.1%) was considered high for this APACHE II level. One plausible explanation for this finding was the profile of our sample. Almost 45.5% of the patients had nosocomial sepsis, 33.5% severe sepsis, the main source of infection was pulmonary (33.5%) followed by abdominal (21%), 25% did not receive a timely intervention (3-h sepsis bundle), and patients were treated in a public health system unit of a low-income country.

The main finding of our study was a 44% reduction in mortality in those who received the 3-h sepsis bundle. Moreover, this intervention was independently associated with survival. We also observed a reduction in ICU admission, as well as a shorter length of stay on these units in patients receiving a 3-h package in wards. Since the publication of the first Surviving Sepsis Campaign Guideline in 2004, some studies have shown a decrease in mortality in patients undergoing sepsis protocols [10, 11]. However, most of these evidences were performed in emergency rooms or intensive care units [12–14]. Hence, one of the main merits of this study was to provide data regarding the impact of a sepsis protocol in wards, mostly the reduction in ICU admissions in a hospital with limited ICU beds.

The low availability of ICU beds in Brazil is a chronic and neglected issue. Most of Brazilian citizens are covered solely by public health system and have access to only 9.9 ICU beds per 100.000 population [15]. Hence, every day, doctors who attend emergency rooms, wards, and post anesthetic recovery rooms have to choice who will get an ICU bed, and patients with sepsis diagnosis have to wait a lot until the ICU admission. The aforementioned concerns and our findings (lower frequency of ICU admissions in bundle group) reinforce the paramount importance of a timely institution of easy interventions, such as antibiotics and fluid replacement, even before the intensivist’s care. Previous evidence has demonstrated that compliance with all bundle metrics is not high [16]. Based on this information and in order to avoid comparisons between different levels of intervention (some individuals with incomplete bundle), our analysis included only those patients submitted to full 3-h bundle.

Most sepsis bundles are based on the early start of antibiotics and fluid resuscitation guided by targets such as central venous pressure and central venous oxygen saturation, for cases with poor tissue perfusion signals. The main reason for this would be the fact that, in recent years, some studies have shown that the aforementioned interventions are correlated with better outcomes in septic patients, even when used alone [13, 14]. To reinforce this hypothesis, the use of sepsis bundles, based on association of early antibiotics with fluid replacement, according to the “Early Goal-Directed Therapy”, has shown positive impact on the outcome of patients with sepsis, with reductions in mortality up to 50% [16, 17]. Our protocol consisted in a standard fluid replacement, based on blood pressure and plasma lactate and early antibiotic therapy, guided by a local infection control committee.

Regarding the limitations of the present study, APACHE II score was applied only at sepsis diagnosis and the impact of different interventions performed in our sepsis bundle was not addressed individually, as well as differences in antibiotics and volume replacement between groups. Furthermore, we did not measure the total volume used in fluid replacement or even parameters such as central venous oxygen saturation or central venous pressure routinely, which makes only speculative the importance of this intervention in our findings. However, in the bundle group, antibiotics were introduced at the time of sepsis diagnosis (within 1st hour) as well as volume replacement. Hence, it is tempting to speculate that the timely institution of these therapies had impact on reducing mortality. This was a single-center retrospective study, which prevents the generalizability of our findings.

## Conclusion

The use of a sepsis bundle in wards was independently correlated with a lower mortality. Moreover, there was a lower frequency of ICU admissions and a shorter length of stay in these units in patients who undergone the 3-h bundle.

## Additional file

Additional file 1: The Statistical Package for the Social Sciences software (SPSS) file. (PDF 26 kb)
