# Supplementary material for: Impact of a sepsis bundle in wards of a tertiary hospital
Source: J Intensive Care. 2017 Jul 18;5:45. doi: 10.1186/s40560-017-0231-2 (PMC5516371; doi:10.1186/s40560-017-0231-2)
Supplement: Additional file 1: — The Statistical Package for the Social Sciences software (SPSS) file. (PDF 26 kb) [file 40560_2017_231_MOESM1_ESM.pdf]

UNIVERSIDADE ESTADUAL DE CIÊNCIAS DA SAÚDE DE ALAGOAS - UNCISAL

Plataforma Brasil

COMPROVANTE DE ENVIO DO PROJETO

DADOS DO PROJETO DE PESQUISA

Título da Pesquisa: Perfil da Sepse em uma Enfermaria de Clínica Médica

Pesquisador: FLAVIO TELES DE FARIAS FILHO

Versão: 2

CAAE: 42247014.2.0000.5011

Instituição Proponente: UNIVERSIDADE ESTADUAL DE CIENCIAS DA SAUDE DE ALAGOAS - UNCISAL

DADOS DO COMPROVANTE

Número do Comprovante: 012813/2015

Patrocionador Principal: Financiamento Próprio

Informamos que o projeto Perfil da Sepse em uma Enfermaria de Clínica Médica que tem como pesquisador responsável FLAVIO TELES DE FARIAS FILHO, foi recebido para análise ética no CEP Universidade Estadual de Ciências da Saúde de Alagoas - UNCISAL em 26/02/2015 às 12:57.
